# Clarkson disease in critically and non-critically ill patients: insights from the Italian IRIS-CLS registry

**DOI:** 10.1007/s11739-025-03890-x

**Published:** 2025-03-08

**Authors:** Riccardo Colombo, Jonathan Montomoli, Teresa Lanzi, Antonella Tosoni, Claudia Agabiti Rosei, Giuseppe Visani, Franco Verlicchi, Chiara Cogliati, Manuela Nebuloni, Maddalena Alessandra Wu

**Affiliations:** 1 Division of Anesthesiology and Intensive Care, ASST Fatebenefratelli Sacco, Luigi Sacco Hospital, University of Milan, Milan, Italy; 2 Division of Anesthesiology and Intensive Care, Infermi Hospital, Romagna Local Health Authority, Rimini, Italy; 3 Division of Internal Medicine, ASST Fatebenefratelli Sacco, Luigi Sacco Hospital, University of Milan, Via G.B. Grassi 74, 20157 Milan, Italy; 4 Pathology Unit, ASST Fatebenefratelli Sacco, Luigi Sacco Hospital, University of Milan, Milan, Italy; 5 UOC 2 Medicine, ASST Spedali Civili Di Brescia, Brescia, Italy; 6 Department of Medical and Surgical Sciences, University of Brescia, Brescia, Italy; 7 Division of Hematology and Stem Cell Transplantation, AST 1, Pesaro, Italy; 8 Transfusion Medicine Faenza-Lugo, Transfusion Service Ravenna, Romagna Health Unit, Ravenna, Italy; 9 Department of Biomedical and Clinical Sciences, University of Milan, Milan, Italy

**Keywords:** Idiopathic systemic capillary leak syndrome, Clarkson disease, Paroxysmal permeability disorders, Capillary leakage, Hypovolemic shock, Hemoconcentration

## Abstract

Idiopathic Systemic Capillary Leak Syndrome (ISCLS) is a poorly understood paroxysmal permeability disorder. This study aimed to characterize the clinical features and severity markers of ISCLS in critically and non-critically ill patients. We analyzed prospectively and retrospectively collected data on ISCLS cases from the Italian IRIS-CLS Registry (January 1995–December 2023). A total of 124 acute episodes were identified in 32 patients; 61 episodes in 26 patients having complete records were included in the analysis. Monoclonal gammopathy was found in 20/23 adults but was absent in all three pediatric patients, who exhibited IgA deficiency instead. At admission, patients presented with hypotension (systolic arterial pressure 87 mmHg [80–105]), tachycardia (110 bpm [96–130]), and hemoconcentration (hematocrit 57% [48–62.7]). ICU admission was required in 70.5%, and cardiac involvement occurred in 49.2% of episodes. Fluid volume infused correlated positively with rhabdomyolysis severity, and the need for renal replacement therapy was more frequent in patients receiving ≥ 3 L. No pharmacologic treatment was effective during the crises. Mortality was 9.8% per episode, and the day-1 Sequential Organ Failure Assessment score was a predictor of death (*p* = 0.0181). Immunoglobulin prophylaxis reduced attack frequency from 0.67 (0.4–1.07) to 0.4 (0.1–0.52) per year (*p* = 0.033). Two adults (8.7%) developed multiple myeloma during follow-up. This study provides a comprehensive overview of ISCLS in both critically and non-critically ill patients. We highlight clinical-biochemical-instrumental red-flags and emphasize the need for increased awareness and early recognition since effective treatments for acute shock remain elusive.

**Supplementary Information:**

The online version contains supplementary material available at 10.1007/s11739-025-03890-x.

## Introduction

Paroxysmal permeability disorders (PPDs) are a heterogeneous group of diseases characterized by a sudden, localized, or generalized increase of capillary permeability [1]. Idiopathic Systemic Capillary Leak Syndrome (ISCLS), also known as Clarkson disease, is a challenging PPD because its pathophysiology is poorly understood, the flare onset is unpredictable, and there is no treatment of proven efficacy for life-threatening attacks.

ISCLS is a rare disease with fewer than 500 cases described worldwide. Although Clarkson described the first case more than sixty years ago [2], awareness of this disease is still poor. Consequently, its real incidence is probably underestimated because it is frequently misdiagnosed as septic shock, fulminant myocarditis, or polycythemia vera.

Usually, ISCLS has a triphasic course [3]. A prodromal phase precedes the crisis by a few days and is characterized by a broad spectrum of symptoms that are variably described in the literature. [3–5] The prodromal phase may break off with restoration to the preceding healthy status or may evolve into the shock phase, which is characterized by abrupt massive plasma extravasation from the intravascular to the extravascular compartment due to loosening endothelial cell junctions resulting in a severe hemoconcentration. The shock phase is caused not only by hypovolemia following plasma shift but also by myocardial dysfunction due to myocardial edema and, sometimes, pericardial effusion [6–8]. These findings are responsible for impaired ventricular filling, which may contribute substantially to the severe circulatory compromise. The shock phase usually terminates in two to three days. At this stage, the recovery phase begins with interendothelial junction restoration, followed by leakage ceasing. Eventually, the extravasated fluids flood the intravascular bed, often leading to hydrostatic pulmonary edema [9]. ISCLS attacks are burdened by high mortality due to refractory mixed (hypovolemic and cardiogenic) shock and the subsequent multiple organ failure [7, 10].

ISCLS patients are asymptomatic during the intervals between crises. No evidence-based therapy exists for this disease because its rarity precludes randomized trials. However, chronic treatment with human intravenous immunoglobulins (IVIG), administered monthly lifetime [11], seems to reduce the frequency and severity of attacks in large cohorts of patients [12, 13] and has been hypothesized that might be helpful in the prodromal or at the beginning of the shock phase in a very limited series [14]. Unfortunately, there are no effective therapies for the shock phase. Aggressive fluid resuscitation can be not only ineffective but also counterproductive: infused fluids extravasate quickly, potentially worsening tissue edema and leading to fluid overload during the recovery phase. Catecholamines or other vasoconstrictors are unable to squeeze the empty vascular bed and may further contribute to tissue hypoperfusion.

The pathophysiological cascade that leads to the sudden increase of endothelial permeability has not been fully clarified. An endothelial hypersensitivity to inflammatory or immune stimuli seems to underlie the vascular leakage [15]. Endothelial cell apoptosis was hypothesized during the crisis [7], but functional adherens complex derangement seems more likely [16]. Respiratory infections are frequently described to precede the ISCLS flare [10, 17, 18]. Recently, it has been demonstrated in vitro that endothelial cells from ISCLS patients are constitutively prone to overexpress nitric oxide (NO) synthesis when exposed to hyperpermeabilizing factors such as vascular endothelial growth factor (VEGF) or histamine [15]. Furthermore, soluble factors may have a role since ISCLS sera collected during acute and intercritical phases have been shown to significantly increase the permeability of human umbilical vein endothelial cells (HUVECs) compared to healthy controls [16, 19]. Most ISCLS patients exhibit monoclonal gammopathy of undetermined significance (MGUS), which, in the context of ISCLS, may be more appropriately defined as “monoclonal gammopathy of clinical significance” (MGoCS) [20], The role of the monoclonal component in the pathophysiology of ISCLS continues to be an active area of research.

The comprehensive and long-term characteristics of ISCLS across repeated crises experienced by both critically and non-critically ill patients remain poorly described.

We conducted a retrospective-prospective study in a large Italian series of ISCLS aiming to assess over three decades its clinical course, signs and symptoms, laboratory findings, effects of the interventions, and severity markers in both critically ill and non-critically ill patients.

## Methods

All ISLS patients admitted to participant centers from 1^st^ January 1995 to 31^st^ December 2023 with available clinical records were retrospectively reviewed. Starting in 2019, all new admissions were prospectively collected into a dedicated electronic database. The study was approved by the local Ethical Committee (Comitato Etico Area 1, Milan, reg. number 0002793 approved on 22^nd^ January 2021).

The criteria for diagnosing ISCLS included the presence of all the following signs: acute hypotension (systolic blood pressure < 100 mm Hg), hemoconcentration (defined as elevated hematocrit or hemoglobin levels exceeding normal values for age and sex or an increase of at least 20% from the patient’s baseline, if available), and development of hypoalbuminemia (albumin levels below the normal range for age and sex). In addition, other causes of secondary capillary leak syndrome or hypoproteinemia were excluded [3, 21, 22].

Clinical data were collected through an electronic collecting platform with pre-specified items in five main areas: (1) demographics and epidemiology, (2) symptoms during the prodromal phase, (3) laboratory findings, (4) imaging, (5) treatment, and (6) outcome.

For the analysis, we considered laboratory data collected at hospital admission and the worst value of hemoglobin, hematocrit, lactate, and serum albumin during the hospitalization. In patients who received IgM-enriched IVIG, we measured IgG and IgM serum concentration every two hours, hematocrit every four hours, and serum albumin every six hours for 24 h from the start of the infusion. To model the fitting curves, we used cubic interpolation between the measured values.

### Statistical analysis

Descriptive statistics were used to characterize the cohort of patients, with denominators reflecting the overall number of patients admitted to the hospital, or those in the ICU, or those with valid data when specified. Continuous variables are shown as median (interquartile, IQR), and categorical variables as numbers (percentage). Categorical data were compared using Fisher’s exact test or a chi-square test. Between-group comparison of continuous variables was accomplished via the Mann–Whitney test or one-way analysis of variance (ANOVA), as appropriate. Comparison of repeated measures within group was assessed with one-way ANOVA, followed by a post-hoc Tukey test. Correlation between non-normally distributed variables was assessed with Spearman’s rho. We fitted both Generalized Linear Models (GLM) and Generalized Additive Models (GAM) to assess the relationship between clinical predictors and mortality risk. The GLM was a logistic regression model with death as the binary outcome and age at the time of crisis, SOFA score on day 1, and cumulative fluids as predictors. For the GAM, we used the same outcome and predictors, but allowed for non-linear relationships by using smooth functions with three knots for each predictor. Both models were fitted using the pooled imputed datasets.

Statistical significance was defined as *p* < 0.05 for a two-tail test. Data were analyzed with SPSS Statistics version 29 (IBM Corp, Armonk, NY) and R version 4.4.0 (R Foundation for Statistical Computing, Vienna, Austria).

## Results

In the study period, a total of 124 acute episodes that occurred in 32 patients were identified. The distribution of new and cumulative cases during the study time is shown in Supplementary Fig. 1. We were able to collect complete records from 61 acute episodes in 26 patients that were considered for the analysis. Demographics of the studied population are shown in Table 1. Fifteen (57.7%) patients were female, 25 (96.2%) were Caucasian, and one (3.8%) was of African ethnicity. The most frequent comorbidity was arterial hypertension. The median age at diagnosis was 54.5 years (35–60), ranging from 3 to 72 years, and three cases were pediatric ISCLS. The median number of attacks experienced was 4 [2–6] per patient, ranging from 1 to 11, and the median number of hospitalizations was 3.5 [2–4] per patient. Serum protein electrophoresis was available in 25 (96.2%) patients, and MGUS was present in 20 (80%) cases, mainly of IgG k type. All pediatric patients had IgA deficiency without MGUS.

**Table 1:** Characteristics of the patients

	Median (IQR) or *n* (%)
Female	15 (57.7%)
BMI	28.2 (25–31.7)
Ethnicity	
Caucasian	25 (96.2%)
African	1 (3.8%)
Age at diagnosis, years	54.5 (35–60)
Age at first crisis, years	54.5 (34.5–60)
Age at admission, years	56.5 (37.5–62.2)
Cumulative number of attacks *per* patient, *n*	4 (2–6)
MGUS†, *n*	20 (80%)
IgG kappa	11 (55%)
IgG lambda	6 (30%)
IgG, not specified	2 (10%)
IgA lambda	1 (5%)
Trigger	
SARS-CoV-2	8 (13.1%)
Other respiratory tract infection	25 (41%)
Other causes	3 (4.9%)
Unknown/not assessed	25 (41%)
Prodromal symptoms and signs (five more frequent), *n*	
Asthenia	24 (39.3%)
Faintness/syncope/dizziness	17 (27.9%)
Oliguria	14 (23%)
Diaphoresis	12 (19.7%)
Abdominal pain	11 (18%)
ICU admission, n	43 (70.5%)
Diagnosis at ICU admission, *n*	
Hypovolemic shock	29 (67.4%)
Septic shock	8 (18.6%)
Prodromal syncope	8 (18.6%)
Cardiogenic shock	6 (14%)
Myocarditis	2 (4.7%)
Pericardial effusion	2 (4.7%)
Other	1 (2.3%)
Not specified	5 (11.6%)
IVIG prophylaxis, *n*	12 (46.2%)
0.5 g/kg	2 (16.7%)
1 g/kg	9 (75%)
2 g/kg	1 (8.3%)
Length of stay in ICU, days	6 (2–10)
Length of stay in hospital, days	11.5 (5.2–21.2)
Death (overall), *n*	6 (23.1%)
Death during SARS-CoV-2-triggered ISCLS crisis, *n*	5 (62.5%)
Number of attacks before IVIG prophylaxis, *n*	3 (2–4)
Number of attacks after IVIG prophylaxis, *n*	2 (0–3)
Mortality rate *per* attack	9.8%

More than one reason per patient was possible

^†^ One missed value. The percentages are calculated on valid data

**Fig. 1:**
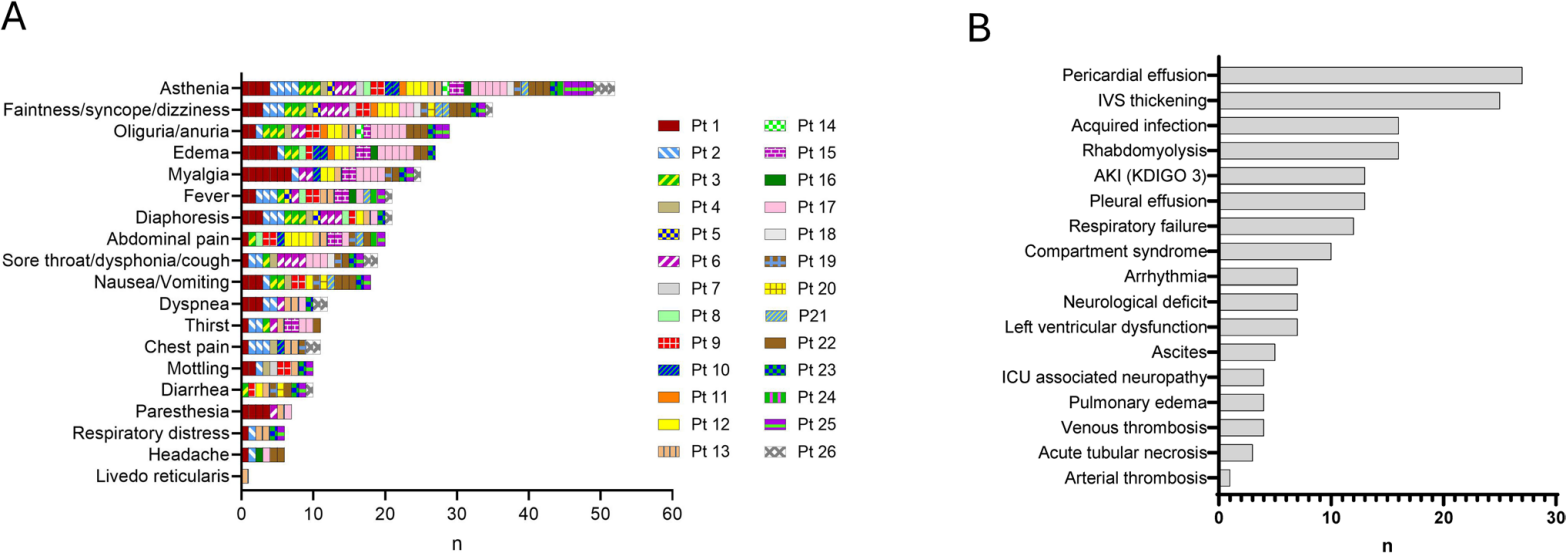
Panel **A**, Characteristics of prodromal symptoms and signs in 61 episodes of ISCLS. Patients tended to experience their own distinctive prodromes with each flare recurrence. Panel **B**, Most common complications occurred during hospitalization. *IVS* interventricular septum, *AKI* acute kidney injury, *KDIGO* kidney disease improving global outcome organization, *ICU* intensive care unit

The most common prodromal symptoms were asthenia, faintness/syncope/dizziness, oliguria, and diaphoresis. Interestingly, each patient had a personal set of symptoms that tended to characterize the further attacks (Fig. 1A).

In 43 (70.5%) episodes, patients were admitted to ICU, with the most common diagnosis of hypovolemic shock (67.4%) followed by septic shock (18.6%).

A trigger was identified in 35 (58.3%) episodes but remained unknown in the others. Respiratory infection was responsible for 32 (91.4%) cases, six (17.1%) of which were due to SARS-CoV-2 (one pneumonia and five upper respiratory tract infections).

Clinical and biochemical parameters at admission and during the flare course are shown in Table 2. At admission, patients were hypotensive (systolic arterial pressure 87 mmHg [80–105]), tachycardic (heart rate 110 bpm [96–130]), with high hematocrit (57% [48–62.7], and lactic acidosis (lactate 3.1 mmol/l [1.6–8.8]). Hemodynamics worsened further during the first 24 h of ICU stay: systolic arterial pressure dropped to 82 mmHg (70–93), *p* < 0.001, heart rate peaked at 121 bpm (110–140), *p* < 0.001, and lactate to 6.3 mmol/l (2.1–10.5), *p* = 0.024. In the meanwhile, hemoconcentration worsened with a hematocrit of 58% (49–65), *p* < 0.001, and serum albumin decreased to 26 gr/l (22–31), *p* = 0.007. In all episodes of ICU admission, patients were oliguric or anuric with a median urinary output of 300 ml (95–650) in the first 24 h (Supplementary Fig. 2). The median time elapsed from admission to the hematocrit normalization as a marker of the duration of the leakage phase was 2 (1–3.5) days. The main treatments and complications during the crises are shown in Table 3 and in Fig. 1B. In 13 episodes (21.3%), patients required renal replacement therapy for a median of 5.5 days (2–10.5).

**Table 2:** Clinical and biochemical parameters at admission and during the flare course

	Median (IQR)
SOFA	3 (2–5)
SAPS 2†	35 (25–51.5)
Systolic arterial pressure, mmHg	
Admission	87 (80–105)
Nadir	82 (70–93)
Mean arterial pressure, mmHg	
Admission	70 (56–80)
Nadir	60 (50–70)
Heart rate, bpm	
Admission	110 (96–130)
Peak	121 (110–140)
Respiratory rate, bpm	20 (16–24.2)
Body temperature, °C	36.0 (36.0–37.1)
Diuresis in the first 24 h, ml	300 (95–650)
Hemoglobin, g/dl	
Admission	19.5 (15.8–21.3)
Peak	20.2 (16.3–22.3)
Hematocrit, %	
Admission	57 (48–62.7)
Peak	58.4 (48.7–64.6)
Leukocytes, cells/μl	16 (8.7–25.8)
Platelets, cells/μl	252 (205–325)
INR	1.1 (1–1.53)
Creatinine, mg/dl	1.4 (1–2.2)
Interval from ICU admission to hematocrit normalization†, days	2 (1–3.5)
Serum albumin, g/l	
Admission	30 (21.2–33)
Nadir	26 (21.7–31)
Creatine kinase, U/l	
Admission	128 (93–237)
Peak	187 (66–8250)
Arterial pH	7.31 (7.11–7.41)
Arterial lactate, mmol/l	
Admission	3.1 (1.6–8.8)
Peak	6.3 (2.1–10.5)
Bicarbonate, mmol/l	15 (12–20.3)
PaO_2_/FiO_2_	351 (236–435)
CRP, mg/l	56 (15–76)
Lactate/albumin ratio	0.08 (0.04–0.37)

*SOFA* sequential organ failure assessment, *SAPS 2* simplified acute physiology score, *CRP* C-reactive protein

^†^Referred to ICU patients only

**Table 3:** Main treatments and complications during the crises

	Median (IQR) or *n* (%)
Bloodletting, *n*	4 (6.6%)
Mechanical ventilation	
* n*	12 (19.7%)
Days	5 (2–10.2)
Renal replacement therapy	
* n*	13 (21.3%)
Days	5.5 (2–10.5)
Catecholamine support	
Norepinephrine, *n*	18 (29.5%)
Norepinephrine, mcg/kg/min	0.39 (0.21–0.55)
Epinephrine, *n*	11 (18%)
Epinephrine, mcg/kg/min	0.2 (0.05–0.5)
Hydrocortisone	
* n*	12 (19.7%)
mg	200 (100–225)
Mechanical circulatory support, *n*	
IABP	2 (3.3%)
VA-ECMO	3 (4.9%)
Specific treatment, *n*	
IVIG	6 (9.8%)
Bevacizumab	1 (1.6%)
Tocilizumab	1 (1.6%)
C1-INH	1 (1.6%)
Methylene blue	1 (1.6%)
IgM-enriched immunoglobulins	3 (4.9%)
Cumulative fluids infused during the shock phase, ml	3000 (1000–6000)
Transfusions, *n*	
RBCs	11 (18%)
FFP	10 (16.4%)
Albumin	22 (36.1%)
Echocardiographic assessment, *n*	40 (65.6%)
Pericardial effusion, *n*	27 (67.5%)
Left ventricular dysfunction, *n*	7 (17.5%)
Interventricular septum thickening, *n*	25 (62.5%)
Pleural effusion, *n*	13 (21.3%)
Venous thrombosis, *n*	4 (6.6%)
Pulmonary edema, *n*	4 (6.6%)
Rhabdomyolysis, *n*	16 (26.2%)
Compartment syndrome, *n*	10 (16.3%)
Fasciotomy, *n*	1 (1.6%)
Acquired infection, *n*	16 (26.2%)

*IABP* intra-aortic balloon pump, *VA-ECMO* veno-arterial extra-corporeal membrane oxygenation, *IVIG* intravenous immunoglobulins, *C1-INH* C1-esterase inhibitor, *RBC* red blood cell (units), *FFP* fresh frozen plasma (units)

The median cumulative volume of fluids infused during the shock phase was three liters (0.98–6.6). It was correlated to the degree of hemoconcentration in terms of maximal hematocrit (*ρ* = 0.423, *p* = 0.016), worst serum albumin (*ρ* = − 0.585, p = 0.007), heart rate (*ρ* = 0.378, p = 0.03), serum lactate (*ρ* = 0.477, *p* = 0.012), lactate to albumin ratio (ρ = 0.768, *p* < 0.001), and severity of rhabdomyolysis assessed by serum creatine kinase levels (*ρ* = 0.589, *p* = 0.005), as shown in Supplementary Fig. 3. Patients who received more than 3 liters of fluids during the shock phase showed an increased need for renal replacement therapy (46.7% vs 10.5%, *p* = 0.018) and a trend toward non-significant higher mortality (31.3% vs 5.3%, *p* = 0.46).

**Fig. 2:**
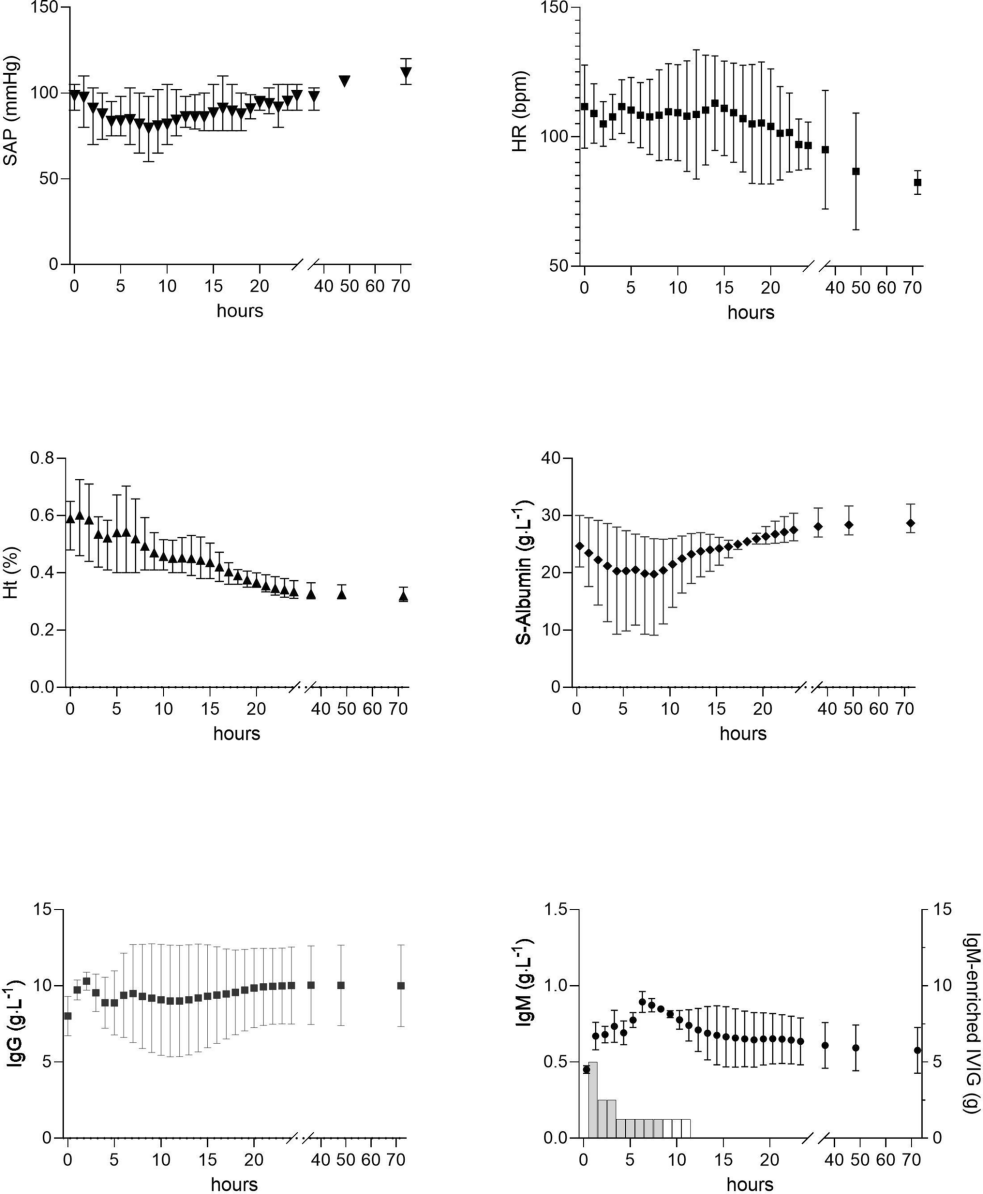
Systolic arterial pressure (SAP), heart rate (HR), hematocrit (Ht), serum albumin, IgG, and IgM concentration changes in three episodes in which the patients received an infusion of 250 mg/kg of IgM-enriched intravenous immunoglobulins according to the following scheme: 5 g in the first hour, 2.5 g per hour in the second and third hour, then 1.25 g per hour until the total dose was reached. Hemodynamics remained compromised at the onset of the infusion, while serum IgM levels showed a transient increase following IVIG bolus administration, but returned to baseline during continuous infusion alongside with hematocrit reduction. Values are displayed as mean ± SEM

In three episodes, IgM-enriched intravenous immunoglobulins (Pentaglobin®, Grifols, Spain) were administered at the dose of 250 mg/kg. The hemodynamics did not improve and, although the levels of serum IgM increased very transiently after the IVIG bolus, they returned to baseline values during continuous infusion (Fig. 2).

In a few crises, empirical therapies were attempted. In one episode, methylthionine chloride (methylene blue) 2 mg/kg was administered intravenously, but the patient worsened further and needed extra-corporeal mechanical circulatory support by venous-arterial Extra-Corporeal Membrane Oxygenation (ECMO) because severe myocardial edema developed. In one episode, bevacizumab for two consecutive days, with no hemodynamic improvement. In one episode, human C1-esterase inhibitor was used without effect.

The most common complication was myocardial involvement, which was due to myocardial edema (pseudohypertrophy detected as transient interventricular septum thickening) and pericardial effusion, both contributing to left ventricular dysfunction (Table 3). Rhabdomyolysis and compartment syndrome occurred in 10 (16.7%) and 7 (11.7%) episodes, respectively, and in 1 case (1.6%) fasciotomy was necessary. Deep vein thrombosis occurred in four (6.7%) episodes.

**Fig. 3:**
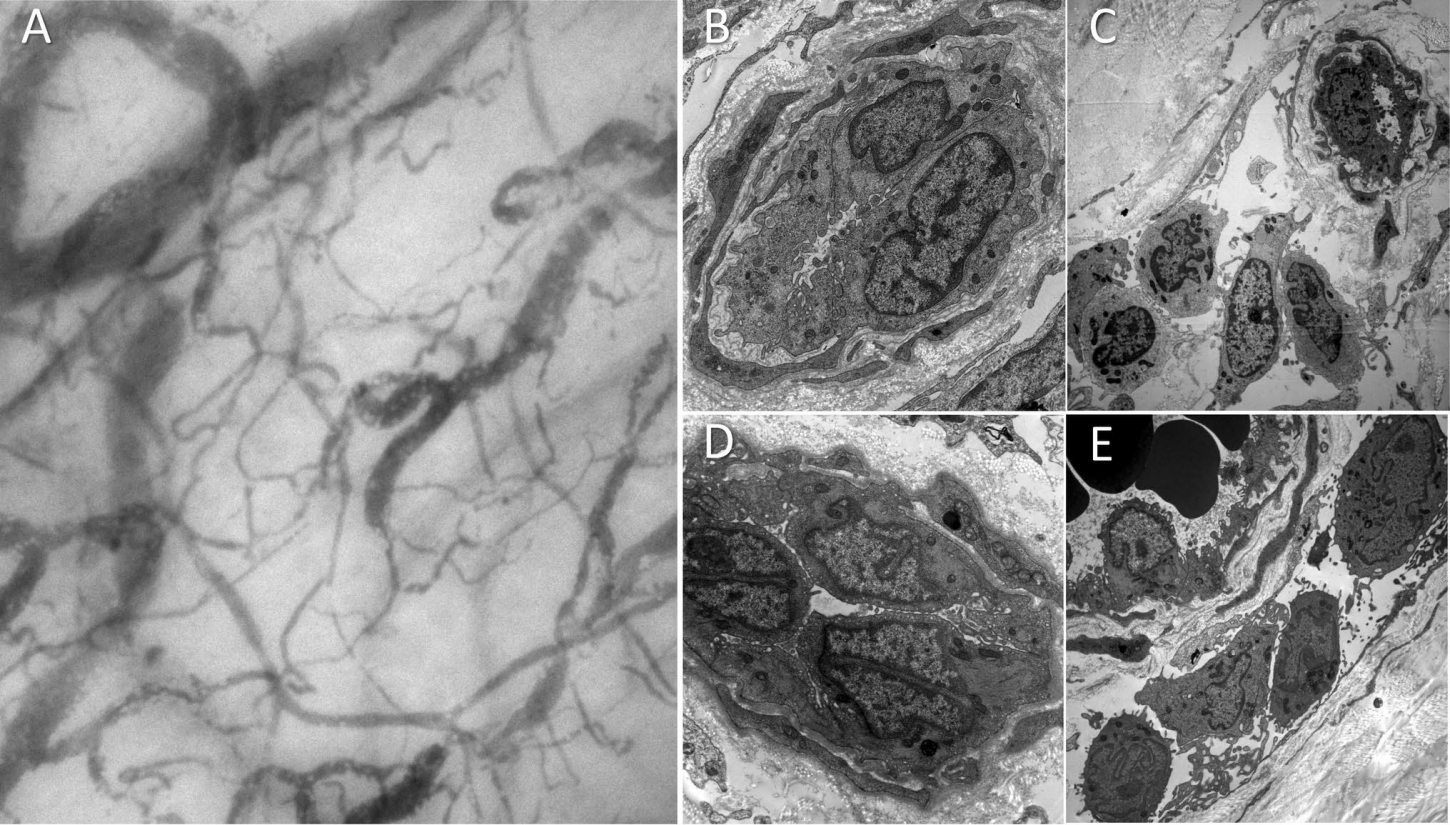
Panel **A** Microcirculatory scan of the sublingual vascular bed during an ISCLS crisis. Despite aggressive fluid resuscitation, capillaries are stuffed with packed red cells and numerous leukocytes (white dots into the capillaries). Dynamic view (available as Supplementary material) shows very low flow or absence of flow in capillaries and small vessels. This is a static image from an in vivo acquired clip of an ISCLS patient. Panels **B**–**E** show electron microscopy (EM) findings of cutaneous biopsies in the abdominal region of ISCLS patients during the intercritical phase (panels **B** and **C**) and during an acute leakage episode with life-threatening shock (panels **D** and **E**). Endothelial cells of cutaneous capillaries have all intact adehrens complexes both in the intercritical and shock phase (panels **B** and **D**), and with surrounding minimal focal chronic inflammatory infiltrate (panels **C** and **E**). OM: × 7000 × 7000; × 3000 × 3000

In one episode, microcirculation was assessed in vivo through CytoCam (Braedius Medical B.V., Huizen, The Netherlands) on the sublingual vascular bed in a mechanically ventilated patient in the ICU. A representative image of microcirculation is shown in Fig. 3A (the video clip comparing this patient to a healthy control is available as Supplementary material). Capillaries and small vessels were stuffed with packed red blood cells and numerous leukocytes with a very slow or even absent flow.

Cutaneous punch biopsies of the periumbilical area were performed during the shock phase on one patient, who eventually died of refractory shock, and on one patient in the intercritical phase. The samples were assessed by both optical microscopy and electron microscopy (EM). No significant alterations were detected at optical microscopy in both patients. At the EM, endothelial junctions were intact in all explored fields, and no signs of endothelial cell apoptosis were found (Fig. 3B-E).

Eighteen patients (69.2%) started IVIG prophylaxis during the study period, and the rate of attacks decreased from 0.67 (0.4–1.07) per year to 0.4 (0.1–0.52) per year, p = 0.033.

Two adult patients among 20 with MGUS (10%) developed multiple myeloma, respectively at the age of 55 and 72 years, after 12 and 13 years from ISCLS diagnosis. They are currently receiving chemotherapy in addition to IVIG prophylaxis. One of them has experienced overall well-being during multiple myeloma treatment and even discontinued IVIG prophylaxis, with only a single mild episode since starting the treatment.

Six patients (23.1%) died, 5 because of refractory shock, and one of prolonged multiple organ failure. In five of them, the trigger was SARS-CoV-2 infection.

At admission, non-survivors compared to survivors had different SOFA scores (10 [2.5–17] vs 3 [2–4], *p* 0.31), SAPS II score (67 [37.7–94.2] vs 29 [22–45], *p* = 0.015), heart rate (140 bpm [112–145] vs 107 bpm [91–126], *p* = 0.025), serum albumin (6 g/l [6–21] vs 30.5 g/l [22.5–33], *p* = 0.045), creatine kinase (679 U/l [155–6491] vs 127 U/l [83–174], *p* = 0.036), pH (7.14 [6.88–7.3] vs 7.34 [7.25–7.42], *p* = 0.045), arterial serum lactate (9.9 mmol/l [9.4–19.9] vs 2.3 [1.4–6.4], *p* < 0.001), whereas during the flare course had higher maximum hematocrit (65.5% [59.5–67.5] vs 58% [48–64], *p* = 0.049) and peak hemoglobin value (22 g/dl [20.7–23.4 g/dL] vs 19.6 g/dl [16–21.9], *p* = 0.047).

Non-survivors received hydrocortisone as a supportive measure during the attacks in five (83.3%) vs eight (14.5%) episodes (*p* = 0.012), mechanical ventilation in five (83.3%) vs seven (12.7%) episodes (*p* < 0.001), and renal replacement therapy in four (66.7%) vs nine (16.4%) episodes (*p* = 0.157).

At the multiple variable analysis assessed by GLM, SOFA score at admission was the only significant predictor of mortality (OR = 1.36 [95%CI 1.05–1.77], *p* = 0.0284). For each one-point increase in SOFA score, the log-odds of death increased by 0.309. This finding was confirmed by the GAM (*p* = 0.0181) which also revealed a linear relationship between SOFA score and the risk of death (Supplementary Fig. 4).

## Discussion

Our study provides one of the most detailed analyses of ISCLS to date, leveraging a uniquely large cohort for such a rare disease. By integrating prospective and retrospective data collected over nearly three decades, we were able to thoroughly investigate disease phases, prognostic factors, and outcomes. Key strengths of our study include rigorous and comprehensive clinical and laboratory assessments, which identified pivotal markers — such as hematocrit and albumin — as early indicators of disease severity and progression.

The number of new diagnoses and recurrent episodes has been increasing in recent years, likely due to greater awareness of the disease among internists, emergency department physicians, intensivists, and hematologists. The median age at diagnosis was in the fifth decade, but it ranged widely from 3 to 72 years, with an almost equal proportion of women and men. MGoCS is an almost distinctive pattern in the adult form of ISCLS. Two patients in our cohort developed a frank picture of multiple myeloma after more than ten years of follow-up.

Our small pediatric cohort exhibited some distinctive features. The absence of a monoclonal component has already been reported in all pediatric cases of ISCLS [22]. Remarkably, we also observed a variable degree of IgA deficiency in all three pediatric patients in our cohort.

We confirmed the finding, previously described by our group in a smaller Italian cohort [4], that prodromal symptoms may vary widely, but each patient tends to have his/her own set of symptoms that characteristically precedes each flare.

Ultrastructural assessment of endothelial cells of cutaneous capillaries on two patients did not find altered adherens complexes or signs of cell apoptosis. Despite the term “systemic capillary leak syndrome” referring to obviously systemic manifestations as shock, our findings endorse the hypothesis that not all endothelial beds are equally affected. Instead, endothelial cell derangement likely involves certain areas more significantly while sparing others. During the prodromal and shock phases, the leakage involves the muscle tissue more than the skin and subcutaneous layers and, in some cases, the serosa, causing muscle tenderness up to compartment syndrome and often pericardial, pleural, or peritoneal effusion.

Of note, we confirmed that myocardial edema may add a cardiogenic component to a primarily hypovolemic shock. In our series, most patients with echocardiographic assessment had myocardial involvement.

Referring to the classical Forrester classification of shock [23], very counter-intuitively, when thinking about capillary leakage, we have to deal with a cold and dry patient. Indeed, mechanical ventilation is primarily used for airway protection in patients who are globally extremely compromised rather than for treating a true respiratory failure, which rarely manifests in the acute phase. Flux across the alveolar-capillary barrier is mainly regulated by the epithelial cells through ionic pumps at the basal membrane side [24], and 92% of the resistance to albumin flux is due to the epithelium [25]. Therefore, pulmonary edema may develop during the post-acute phase due to rising hydrostatic pressure because of the reverse passage of fluids from the extravascular into the intravascular space. This phenomenon may be exacerbated by impaired relaxing of the left ventricle due to transient myocardial edema and pericardial effusion.

Similarly to what is observed for the pulmonary district, consciousness is usually preserved even during advanced shock states given that cerebral edema is rare and sometimes appears to develop not because of a primary involvement of the cerebral vascular bed but rather as an accompanying complication of ischemic stroke, probably stemming from a pro-thrombotic milieu.

The philosophy of “damage control” with careful use of the “minimal effective dose” of fluids still seems to be the most appropriate one and the most challenging target, as excessive fluid replacement is correlated with increased morbidity in our series and was shown to be associated with mortality in the EureClark Registry [10]. Importantly, the use of noninvasive handheld video microscopy in one patient revealed that, despite aggressive fluid resuscitation, the microcirculatory flow remained “paralyzed”.

Our findings further confirm the role of IVIG prophylaxis, highlighting its undeniable but sometimes insufficient efficacy for flare prophylaxis. In this non-randomized, non-controlled observational study, the rate of ISCLS attacks per patient decreased after the start of IVIG prophylaxis. However, some patients died despite IVIG prophylaxis. The fatal crisis occurred a few days before the scheduled IVIG infusion at the dose of 1 g/kg every 30 days. It is unknown (even though suggested empirically by some authors) whether a higher dose or shortening the intervals between doses during periods at higher risk of viral respiratory infection, such as seasonal flu epidemics or SARS-CoV-2 waves, might be beneficial.

Our study does not provide sufficient data to draw conclusions about the use of IVIG during ISCLS attacks.

Despite experimental evidence of the involvement of the NO pathway in the pathophysiology of ISCLS and the intrinsic hyper-responsiveness of ISCLS-derived endothelial cells [15], the role of the NO pathway in ISCLS, both during the intercritical and acute phases, remains under investigation. In addition, NO-mediated reactive hyperemia has been shown not to be impaired in ISCLS patients in vivo [26]. Similarly, the blockade of NO synthesis by methylene blue in an episode from this series did not change the course of the crisis, as previously reported in both acute and chronic forms of ISCLS [4, 27].

Other empirical therapies for acute flares, such as bevacizumab, and C1-esterase inhibitor, remain anecdotal and were ineffective in the cases described.

While our study includes a substantial number of patients and episodes for such a rare condition, it is important to acknowledge that the small sample size limits our ability to conduct robust inferential analysis on risk and prognostic factors related to mortality. Nonetheless, our findings should be seen as a ‘call to action’. Although ISCLS is rare, it is likely underdiagnosed due to limited awareness among physicians. Furthermore, the lack of formal recognition of this potentially fatal disease complicates the prescription of immunoglobulins for prophylactic purposes, particularly in smaller centers that may be unfamiliar with the complex bureaucratic procedures required to secure reimbursement for patients on monthly IVIG prophylaxis.

The longitudinal design of our study has provided insights into the potential evolution of MGUS into multiple myeloma, as documented in two cases after more than a decade of follow-up. These findings underscore the need for prolonged monitoring and support the emerging concept of monoclonal gammopathy of clinical significance in the context of ISCLS.

## Conclusion

Our study outlines the features of ISCLS in an Italian cohort, encompassing both critically ill and non-critically ill patients. ISCLS is frequently misdiagnosed, making its timely recognition essential for appropriate management. Although no specific biomarker is currently available, a simple laboratory test such as hematocrit may serve as the earliest marker for each phase. Hematocrit acts as a ‘red flag’ at the onset of capillary leakage, with levels rising due to plasma extravasation and typically peaking within a day. The subsequent reduction in hematocrit reflects the shifting of fluids back into the intravascular compartment, which may indicate the restoration of interendothelial junction function. Identifying the transition from the acute to the post-acute phase is critical, as the therapeutic strategy must change significantly between these phases.

Our findings support the value of lifelong monthly IVIG prophylaxis. While research into novel targeted therapies is still ongoing, the primary approach during the acute phase remains one of “damage control,” involving cautious fluid administration.

Collectively, our results offer a solid foundation for improving the understanding, monitoring, and management of this rare condition, while providing a framework for future research and the development of clinical guidelines.

## Supplementary Information

Below is the link to the electronic supplementary material.

Supplementary file1 (MP4 720 KB)

Supplementary file2 (PDF 1360 KB)

## Data Availability

The data will be available upon reasonable request.
