# Supplementary material for: Clarkson disease in critically and non-critically ill patients: insights from the Italian IRIS-CLS registry
Source: Intern Emerg Med. 2025 Mar 8;20(4):991–1001. doi: 10.1007/s11739-025-03890-x (PMC12130070; doi:10.1007/s11739-025-03890-x)
Supplement: Supplementary file 2 — Supplementary file2 (PDF 1360 KB) [file 11739_2025_3890_MOESM2_ESM.pdf]

Clarkson Disease in Critically and Non-Critically Ill Patients: Insights from the Italian IRIS-CLS Registry.

Supplementary Figures

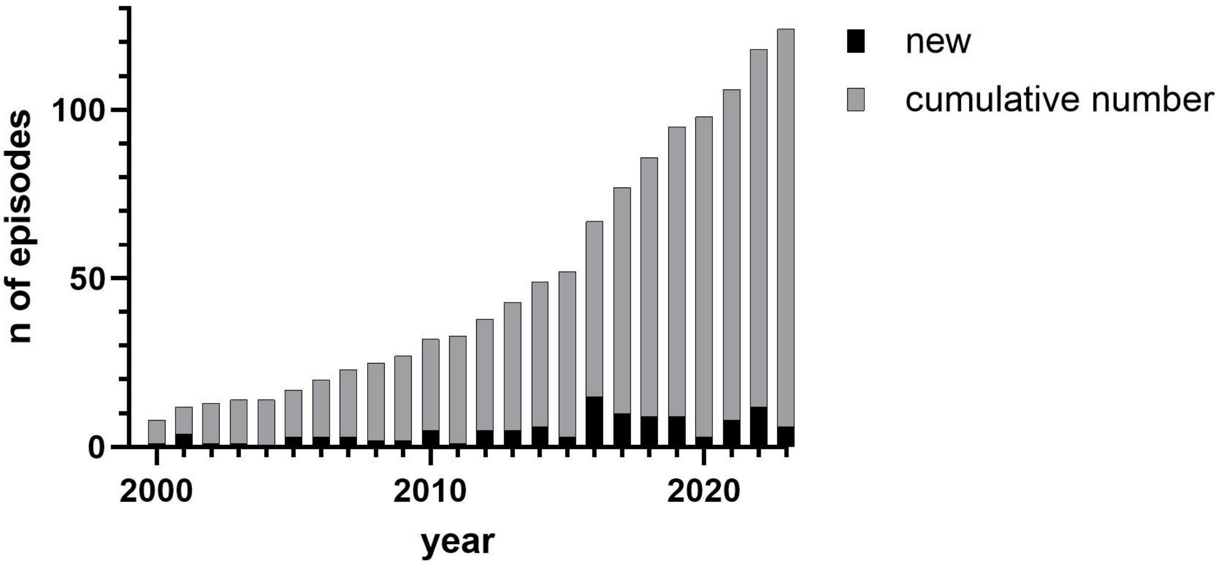

Supplementary Fig. 1 Number of new episodes and cumulative number throughout the study period. Seven episodes occurred between 1995 and 1999.

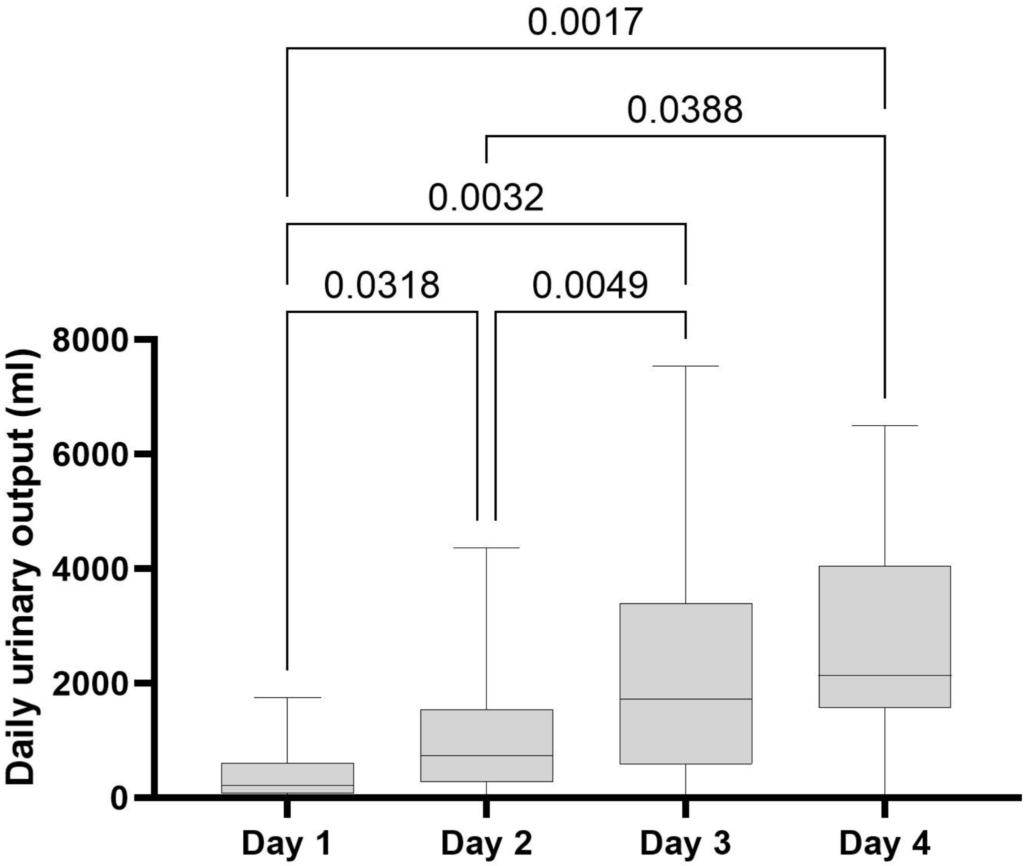

Supplementary Fig. 2 Daily urinary output in severe episodes needing ICU admission.

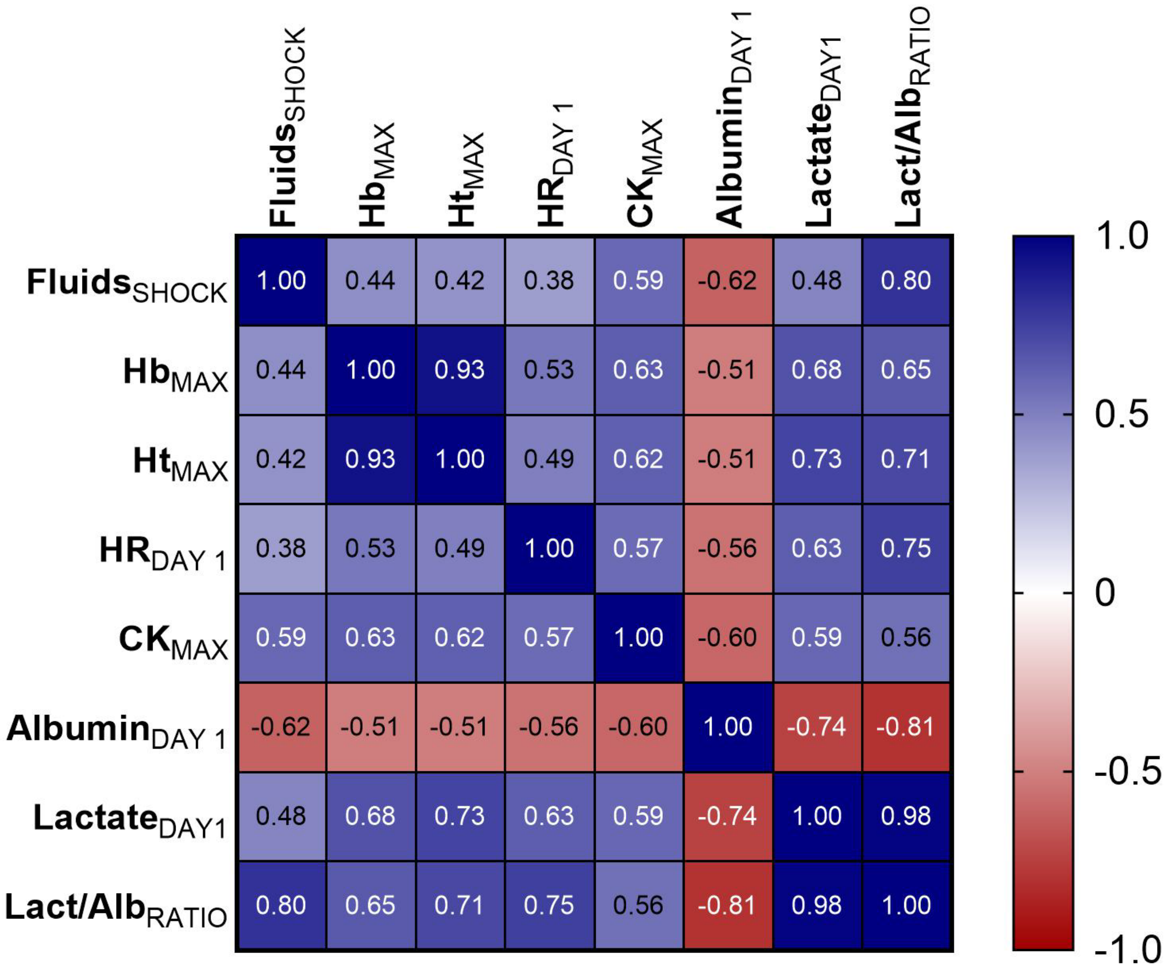

Supplementary Fig. 3 Heatmap matrix of correlation. Values displayed are Spearman's ρ.

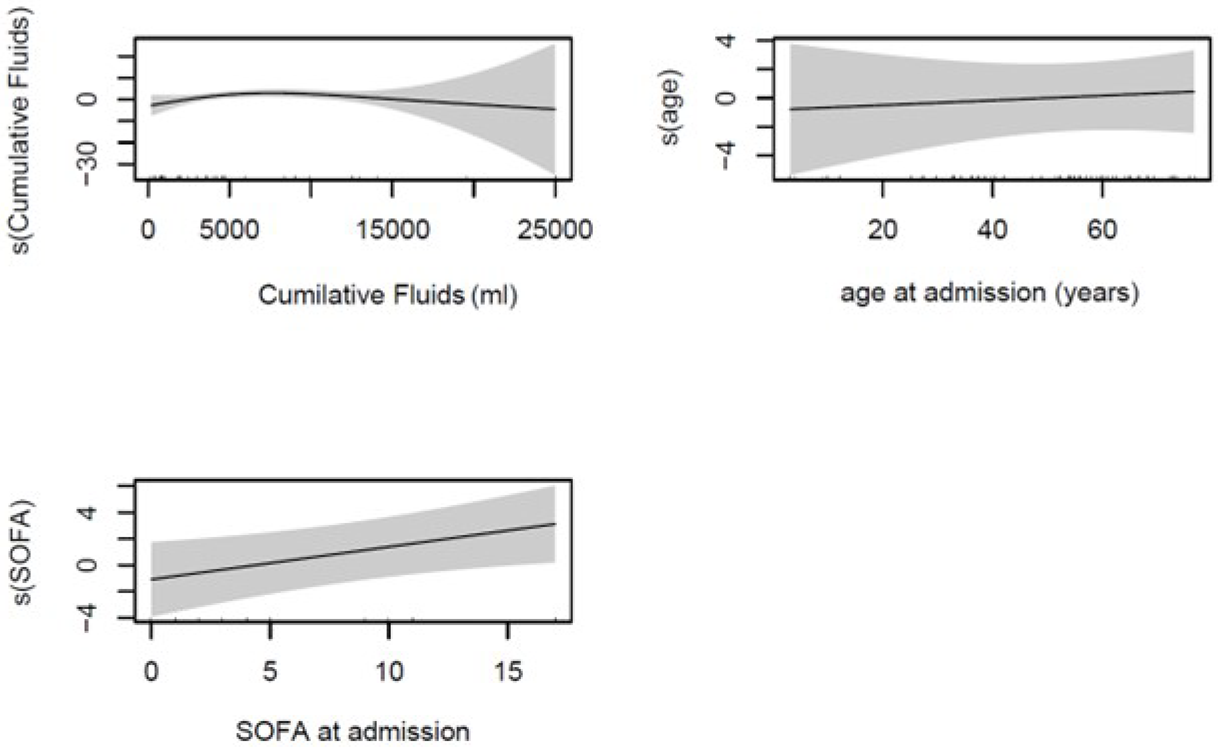

Supplementary Fig. 4 Generalized Addictive Model for mortality. Cumulative fluids: Effective Degrees of Freedom (edf) = 1.843, p = 0.0945; Age at admission: edf = 1.00, p = 0.6841; SOFA at day of admission: edf = 1.00, p = 0.0181. Model fit: R-squared = 0.311, Deviance explained = 39%.
